# Black Americans Train Clinicians Who Provide Care to Older Black Americans: Program Development and Pilot Outcomes

**DOI:** 10.1093/geroni/igaf122.1285

**Published:** 2025-12-31

**Authors:** Ronit Elk, Shena Gazaway, Alvin Reaves, Michael Barnett

**Affiliations:** The University of Alabama at Birmingham, Birmingham, Alabama, United States; School of Nursing, Birmingham, Alabama, United States; US Acute Care Solutions, Rockville, Maryland, United States; Four Seasons Hospice, Flat Rock, North Carolina, United States

## Abstract

Lack of appreciation of cultural differences during serious illness compromises care for seriously ill older African American patients. Training programs for clinicians in providing culturally appropriate care for older adults of different cultures is lacking. The goals of this study were to partner equitably with a southern African American community to create a training program for clinicians on how to communicate in a culturally congruent and respectful way with older southern, African Americans with serious illness and at end of life, and to test program delivery feasibility. Community Based Participatory Research guided the equitable partnership. The Community Advisory Board (CAB) guided the creation and implementation of two focus groups (caregivers and pastors). Based on focus groups’ results on important cultural values and respect for lived experiences, the CAB created 4 training videos. Each video highlighted a key message for clinicians- cultural, religious, and demand for equal care. The videos were incorporated into a training program based on Kolb’s Adult-based Experiential Learning Theory, and a training guide created for the 3-hour training that can be delivered via zoom or in person. Donabedian’s Structure-Process-Outcome model was used to determine program delivery feasibility. Two pilot training sessions were held, with high rate of clinician attendance. At three months post-training clinicians reported a high rate of adhering to the community recommendations. The first training program developed by African American community members to train clinicians was feasible to create and implement, and resulted in clinicians changing their practice to adhere to the community’s recommendation.

